# Acyl-Homoserine Lactone Quorum Sensing in the *Roseobacter* Clade

**DOI:** 10.3390/ijms15010654

**Published:** 2014-01-07

**Authors:** Jindong Zan, Yue Liu, Clay Fuqua, Russell T. Hill

**Affiliations:** 1 Institute of Marine and Environmental Technology, University of Maryland Center for Environmental Science, 701 E Pratt St., Baltimore, MD 21202, USA; E-Mails: jzan@wisc.edu; 2 Department of Chemistry, University of Wisconsin-Madison, 1101 University Ave, Madison, WI 53706, USA; E-Mail: yliu@chem.wisc.edu; 3 Department of Biology, Indiana University, 1001 E. 3rd St., Jordan Hall 142, Bloomington, IN 47405, USA

**Keywords:** Quorum sensing, signaling, symbiont, LuxI homologue, *Ruegeria*, *Phaeobacter*, *Dinoroseobacter*

## Abstract

Members of the *Roseobacter* clade are ecologically important and numerically abundant in coastal environments and can associate with marine invertebrates and nutrient-rich marine snow or organic particles, on which quorum sensing (QS) may play an important role. In this review, we summarize current research progress on roseobacterial acyl-homoserine lactone-based QS, particularly focusing on three relatively well-studied representatives, *Phaeobacter inhibens* DSM17395, the marine sponge symbiont *Ruegeria* sp. KLH11 and the dinoflagellate symbiont *Dinoroseobacter shibae*. Bioinformatic survey of *luxI* homologues revealed that over 80% of available roseobacterial genomes encode at least one *luxI* homologue, reflecting the significance of QS controlled regulatory pathways in adapting to the relevant marine environments. We also discuss several areas that warrant further investigation, including studies on the ecological role of these diverse QS pathways in natural environments.

## 1. Introduction

The *Roseobacter* clade is a diverse group of bacteria that belong to the *Rhodobacteriaceae* family of the *Alphaproteobacteria*, and its members, including 17 different genera, share greater than 89% *16S rRNA* gene sequence identity [1,2]. Roseobacterial members have a diverse and broad ecological distribution but are exclusively restricted to marine or hypersaline environments [2,3]. Roseobacters have been characterized as ecological generalists and exhibit different lifestyle strategies, including heterotrophy, photoheterotrophy and autotrophy [3,4]. These bacteria are numerically abundant and are estimated to account for about 20%–30% of the bacterial *16S rRNA* genes in ocean waters [2,5]. Furthermore, many members are found to be associated with marine invertebrates (such as marine sponges or corals), marine algae, dinoflagellates and sea grasses [6–9]. Several rosobacterial strains have been reported to cause diseases in marine invertebrates or algae [5]. An important ecological function of members in this clade is to participate in global sulfur cycling through metabolizing dimethylsulfoniopropionate (DMSP), an abundant organic sulfur compound produced by marine phytoplankton [10]. The pathways and enzymes participating in the demethylation or cleavage pathways have been comprehensively reviewed by Moran *et al.* [10].

It is now well accepted that most bacteria have chemical communication systems that allow signaling and response between individuals and thus can coordinate collective behaviors. Among these systems, quorum sensing (QS), a process by which bacteria sense and perceive their population density through the use of diffusible signals has been extensively studied in many bacterial species and the progress of the research on QS has been comprehensively reviewed [11–13]. A well-established form of QS among the Proteobacteria relies on acylated homoserine lactone (AHL) signal molecules.

## 2. General Mechanisms of AHL Quorum Sensing

Signaling via AHLs involves enzymes that synthesize the molecular cues, AHL synthases, and response pathways including signal receptors that perceive these cues and directly or indirectly transduce the response to changes in bacterial behavior. The first of these systems to be identified was in the marine vibrios, and the production and response to AHLs was extensively studied in *Vibrio fischeri*, a bioluminescent symbiont of marine fishes and squids. Bioluminescence in *V. fischeri* is regulated by production of the 3-oxo-hexanoyl-homoserine lactone (3-oxo-C6-HSL), so that only the dense populations associated with marine animals produce light. The enzyme that is responsible for AHL synthesis signals is called LuxI due to its role in bioluminescence control [14]. For sparse populations of *V. fischeri*, the concentration of the AHL is low and it can quickly transit across the bacterial cell membrane by passive diffusion to be diluted in the environment. As the population density increases, the AHL molecules accumulate proportionally. Once the population density and thus the concentration of the AHL reach a certain threshold concentration at which it binds to its cognate receptor, the AHL-responsive transcription factor called LuxR, a cytoplasmic protein. The complex of LuxR and AHL turns on or turns off a certain set of genes and thus coordinates the group behavior, including bioluminescence [13]. Many other bacteria are now known to utilize one or more LuxI–LuxR-type QS systems and AHL signal molecules. The range of QS-regulated phenotypes in these diverse quorum sensing bacteria extends well beyond bioluminescence and includes exoenzyme synthesis, antibiotic production, virulence factor elaboration, horizontal gene transfer, motility, and biofilm formation [11].

**Figure 1.**
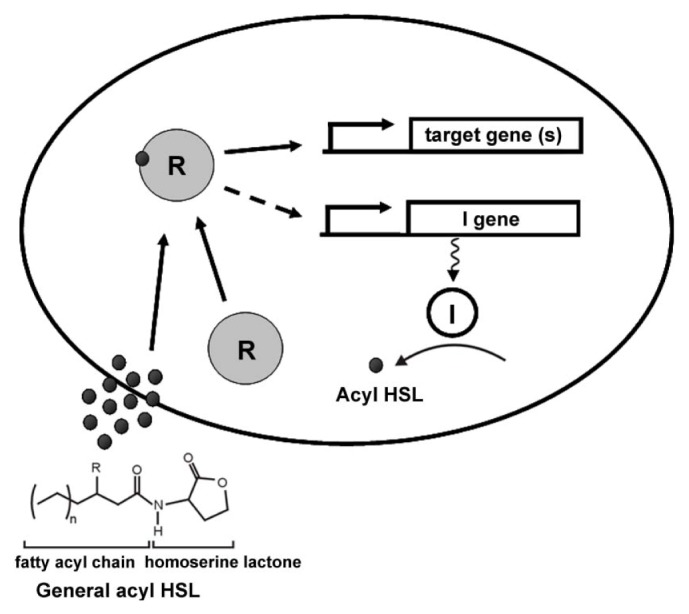
Basic model of quorum sensing (QS) circuits. The eclipse represents a cell. The *I* gene represents the *luxI* homologue. R represents the acylated homoserine lactone (AHL) receptor LuxR protein. The dark unfilled circle represents the LuxI enzyme while the dark solid dots represent the AHL molecules. Stalked arrows indicate the transcription of the genes and the dotted line with arrow shows the positive feedback by the complex of the LuxR receptor and AHLs on the AHL synthase gene. The solid line with arrow depicts the function of R complex on the target genes. Squiggly line indicates translation of *I* gene and solid curved line with arrow indicates enzymatic function of *I* gene. The left corner shows the basic structure of a typical AHL molecule. *N* can vary from 4 to 18. *R* can be oxo, OH or H.

All AHL molecules have a homoserine lactone linked to a fatty acid chain via an amide bond. The chain length can vary from 4 to 18 carbons and the oxidation status of the third carbon can vary from fully reduced to fully oxidized (Figure 1). The substrates for LuxI-type enzymes are *S*-adenosylmethionine (SAM) and fatty acyl precursors conjugated to the acyl carrier protein (ACP). The methionine in SAM provides the homoserine moiety, which is conjugated to the acyl chain donated by acyl-ACP [15,16]. Most LuxI-type enzymes are roughly 180–230 amino acids (aa) in length although in some cases can be longer (several such examples are found among the roseobacters) [4,17]. The LuxI-type protein can be divided into *N*-terminal and *C*-terminal regions. The *N*-terminal region, responsible for interactions with the substrate SAM, is the most conserved part of the enzyme and has eight invariable residues, Arg24, Phe29, Trp35, Asp46, Asp49, Arg70, Glu100 and Arg103 (the numbering is based on *V. fischeri* LuxI) [18]. In contrast, there is relatively low conservation in the *C*-terminal region, which is involved in the recognition of the more variable acyl chain, and the acyl-ACP fatty acid biosynthetic intermediate [18].

In many bacterial systems, the LuxI homologue is genetically linked with a cognate LuxR homologue, encoding the AHL receptor [19]. Most LuxR-type proteins are transcriptional activators that multimerize when associated with an AHL, and bind to sequences upstream of their target genes. The *N*-terminal domains of LuxR-type proteins contain the AHL-binding sites and the *C*-terminal domains encode the DNA binding motif. These two domains are linked together via a conformationally flexible linker. Binding to the cognate AHL stimulates the LuxR receptor to multimerize, and can also enhance the resistance to protease-mediated degradation of the LuxR-type protein [20–22]. The *C*-terminal domain of LuxR-type proteins contains a Helix–Turn–Helix motif (HTH) that is able to recognize a conserved palindromic DNA sequence element 18–22 bp in length and named the *lux-*type box upstream of target genes [23]. Often, *luxI* homologues have a *lux*-type box in their promoter region, thus their cognate LuxR protein can also stimulate the expression the AHL synthase, resulting in greater AHL synthesis and creating a positive feedback loop to amplify and perhaps insulate the QS response once engaged (Figure 1). In a small subset of LuxR-type proteins, the apoprotein that is not associated with AHL is the multimeric form and is proficient to bind DNA, and interaction with the ligand causes dissociation and inactivation [24].

LuxI and LuxR from *V. fischeri* represent one of the best characterized QS models, but this basic mechanism is now known to be widespread among diverse Proteobacteria, controlling a range of target functions including biofilm formation, motility, production of virulence factors, antibiotic synthesis and horizontal gene transfer. A large fraction of QS research has focused on animal and plant pathogens, such as *Pseudomonas aeruginosa*, *Vibrio cholerae*, *Vibrio harveyi*, *Agrobacterium tumefaciens*, *Serratia* spp. and *Erwinia* spp. Among marine bacteria the vast majority of studies have focused on *Vibrio* spp., with far less attention to other major marine bacterial groups.

Recently, however, there has been significant progress on the predominantly nonpathogenic *Roseobacter* clade, with insights into QS mechanisms that utilize AHLs as well as other signals. This review is aimed at summarizing the current understanding of QS pathways in this abundant and ecologically important marine bacterial clade and discussing future research. Much of our discussion focuses on recently characterized QS pathways in *Phaeobacter inhibens* DSM17395 (formerly known as *P. gallaeciensis* DSM17395) [34], the marine sponge symbiont *Ruegeria* sp. KLH11 and the dinoflagellate symbiont *Dinoroseobacter shibae* DFL-12^T^ (Table 1). In addition a comprehensive survey of the LuxI homologues in roseobacterial species from available genome sequences is included with roughly 80% of roseobacterial species encoding LuxI homologues, suggesting AHL mediated regulation plays important roles for this diverse and abundant bacterial clade in adapting to different niches in the marine environment.

## 3. AHL Production in the *Roseobacters*

Niches in the marine environments that contain high nutrient concentrations would be conducive to supporting dense bacterial populations that might reasonably be expected to utilize AHL-based QS. These environments include marine snow, invertebrates, macro- and microalgae, and plants. Gram *et al.* [35] screened bacteria isolated from marine snow for AHL production and showed that three roseobacterial isolates are able to produce AHL molecules detected by an *A. tumefaciens* AHL biological reporter system. Marine snow is made up of organic and inorganic particles and is rich in energy sources and nutrients. Bacteria can colonize marine snow and produce exoenzymes to degrade and catabolize the nutrient sources available in the aggregated organic particles [35].

Bruhn *et al.* [36] found that the strain *Phaeobacter* sp. 27-4 isolated from turbot rearing facility produced 3-hydroxyl-decanoyl-homoserine lactone (3-OH-C10-HSL). Taylor *et al.* [37] reported a bacterial strain in the genus *Ruegeria* isolated from the Australian marine sponge *Cymbastela concentrica* that produced AHLs detected by several AHL reporter systems sensitive to short acyl chain (C4–C6) length AHLs (based on *Chromobacterium violaceum*) and longer acyl chain (C6–C14) length AHLs (based on *A. tumefaciens*). In the same study, organic extracts from 27 of 37 different marine invertebrates including marine sponges, corals, ascidians and bryozoans stimulated the short-chain AHL reporter, indicating the presence of bacteria producing short-chain AHLs associated with these invertebrates. The specific AHLs responsible for induction of the reporter systems in these studies by Gram *et al.* [35] and Taylor *et al.* [37] were not chemically identified.

Wagner-Döbler *et al.* [1] screened 102 marine bacterial strains isolated from several different marine environments for AHL production using a mass spectrometric approach in parallel with biological reporters and found that 31 roseobacterial strains out of 67 alphaproteobacterial strains that were detected were able to significantly increase the fluorescence of their AHL reporter system, suggesting AHL production. The majority of these AHL-producing roseobacterial strains were isolated from either marine dinoflagellates or picoplankton. Subsequently, Mohamed *et al.* [6] reported that isolates from the *Silicibacter-Ruegeria* (SR) subgroup of the *Roseobacter* clade are the dominant AHL producers from bacterial isolates associated with two marine sponges, *Mycale laxissima* and *Ircinia strobilina*. These roseobacterial symbionts produce a mixture of short and long-chain length AHLs revealed by thin layer chromatography (TLC) coupled with a series of AHL biological reporters covering a range of AHL acyl chain length (C4–C16). Using similar methods, we have screened over 400 bacterial strains isolated from the same two sponges collected over three consecutive years and have found SR roseobacters to represent close to 75% of the AHL-producing isolates and the remaining AHL positive isolates were dominated by marine vibrios [38].

## 4. QS in Phaeobacter inhibens DSM17395

A *P. inhibens* strain DSM 17395 was isolated from the Atlantic coast near northwestern Spain [25]. The LuxI homologue *pgaI* in this strain synthesizes 3-hydroxydecanoyl-HSL (3-OH-C10-HSL) and the cognate LuxR homologue is called *pgaR*. There seems to be a minor effect of PgaR on the expression of *pgaI* [26]. Initially, it was found that null mutants of *pgaI* and *pgaR* cannot synthesize tropodithietic acid (TDA), an antibiotic produced by several members in the *Roseobacter* clade, and also fail to produce a yellow-brown pigment, when grown in marine broth with shaking for 24 h [26]. However, recent studies show that different carbon sources determine the role of QS in regulating the production of TDA [28] and the production is only delayed in QS mutants in agitated culture in marine broth [27]. More importantly, QS is not important for the TDA production when *P. inhibens* DSM17395 is used as a probiotic treatment in aquaculture system [27]. Interestingly, the antibiotic TDA itself may also be considered a signal molecule in *P. inhibens* DSM 17395. Addition of exogenous TDA into QS mutants increases pigment production and induces the expression of its own synthesis genes [26]. Support for the proposal that TDA is a QS molecule also comes from studies with *Silicibacter* sp. TM1040, a roseobacterial symbiont of dinoflagellates. Addition of TDA to *Silicibacter* sp. TM1040 culture also increases the expression of TDA synthesis genes most likely via TdaA [39]. This type of internal positive feedback indeed resembles that of many LuxI homologues in response to AHLs via their cognate LuxR proteins.

## 5. QS in the Marine Sponge Symbiont *Ruegeria* sp. KLH11

**Figure 2.**
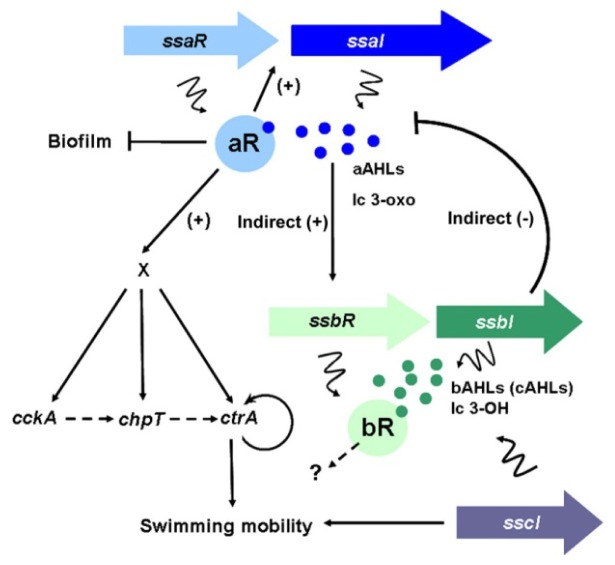
Interconnected QS network and model for *ssaRI* and *cckA-chpT-ctrA* regulatory circuit that controls KLH11 flagellar motility. Lengths of the *ssaRI*, *ssbR*, *and sscI* genes are drawn in scale. Genes and products are colored in corresponding colors, with R-genes more darkly colored and I-genes lighter colored. The dark blue dots represent the AHLs, mainly long chain (lc) 3-oxo-HSLs, synthesized by SsaI. The dark green dots represent the AHLs, mainly long chain (lc) 3-OH-HSLs, synthesized by SsbI. Lines with bars indicate inhibition and arrows indicate activation. Squiggly lines indicate translation of genes or products of enzyme action. The dashed line with arrows indicates the potential phosphate flow from CckA to CtrA via ChpT. The curved lines with arrows around CtrA indicate positive feedback loops. The “X” indicates the unknown regulator(s). The figure is modified from references [17] and [29].

We have been examining *Ruegeria* sp. KLH11 (hereafter referred as KLH11) as a representative to study QS pathways in marine roseobacters. Arguably, KLH11 represents one of the best-studied roseobacterial species for its AHL-mediated QS circuits. Our studies have shown that KLH11 has very complex and interconnected QS networks (Figure 2). KLH11 has two LuxI–LuxR pathways and one LuxI solo, designated *ssaIR*, *ssbIR* and *sscI*, respectively. SsaIR are orthologous to SilI1R1 in *Ruegeria pomeroyi* DSS-3 while SsbIR are orthologues of SilI2R2 [4,17].

SscI, which is absent *in R. pomeroyi* DSS-3, shares *ca*. 80% identity on the amino acid level to SsbI. Sequence conservation between the *ssbI* and *sscI* genes is strikingly confined to the coding sequences, without similarity in their flanking regions, suggesting that *sscI* is the result of a relatively recent gene duplication event from *ssbI*. Furthermore, the adjacent upstream regions that surround *sscI* are checkered with several transpose and phage integrase genes, suggesting a high local level of chromosomal rearrangement [40].

All three *LuxI* genes can synthesize AHLs when expressed both in *Escherichia coli* and KLH11. Each of the three LuxI-type enzymes drives synthesis of several different long chain AHLs when expressed in KLH11 and in the heterologous *E. coli* (both odd and even-numbered acyl side chain lengths can be detected). SsaI is biased towards synthesis of relative long chain 3-oxo-AHL derivatives (C12–C16) whereas SsbI and SscI tend to direct synthesis of long chain 3-hydroxylated AHLs (C12–C14), although these specificities are not absolute. Interestingly, wild type laboratory cultures of KLH11 mainly contain AHLs derived from SsbI or SscI, both of which synthesize an indistinguishable spectrum of AHLs [17,40]. However null KLH11 *ssaI* mutants do not produce detectable AHLs, suggesting that although SsaI-derived AHL levels are low in wild type cultures, their activity is required to produce SsbI and SscI-derived AHLs. SsaI-derived AHLs also seem to play a major role in regulating motility and biofilm formation [17,29]. Generally, long chain AHLs are more stable in alkaline environments (the average pH in ocean is *ca.* 8.2) compared to short chain AHLs [41,42]. Production of long chain length AHLs has also been reported in several other roseobacters [1]. Thus, it is possibly characteristic of roseobacters to produce long chain length AHLs, compounds with a relatively long half-life in the marine environment.

SsaR activates expression of *ssaI* in response to SsaI-directed AHLs, hence manifesting a positive feedback loop in the SsaRI system. A sequence upstream of *ssaI* gene is required for this activation; although it lacks homology to any known *lux*-type box, and is not an inverted repeat as are many LuxR-type proteins DNA binding sequences. In contrast SsbR does not activate the expression of *ssbI*. Similar to *ssbI*, the *sscI* solo gene with no genetically linked LuxR homologue is not regulated by either SsaR or SsbR [17,40].

Flagellar motility in KLH11 is strictly dependent on the SsaRI system [17,29]. SsaR and long chain AHLs are required, and indirectly activate the expression for *cckA*, *chpT* and *ctrA* genes, which in turn control flagellar motility gene expression. CckA is a hybrid two-component type sensor histidine kinase with its own internal receiver domain, including a phosphorylation site that contains a conserved aspartate [43]. ChpT is single domain histidine phosphotransferase (Hpt) protein, and CtrA is a response regulator with a conserved aspartyl receiver domain and a separate DNA binding domain. This pathway is well conserved throughout the Alphaproteobacteria and commonly controls motility, but outside of the *Rhodobacteriaceae* is frequently essential due to crucial roles in the regulation of cell division [44]. Among KLH11 and other roseobacters the CckA-ChpT-CckA pathway is non-essential, but controls flagellar motility. The intermediate regulator(s) that connects SsaRI and *cckA*-*chpT*-*ctrA* phosphorelay system remains to be identified [29]. The SsbRI system does not affect flagellar motility in KLH11 [17], whereas *sscI* does contribute, at a modest level [40].

Biofilm formation increases significantly in *ssaI* and *ssaR* mutants compared to wild type KLH11. Despite their lack of motility these mutants accumulate more rapidly on surfaces. Flagellar motility contributes to biofilm formation in many bacterial species. It is plausible that the effect of QS on biofilm formation in KLH11 is strictly due to the loss of flagellar motility. However, an *ssaIfliC* double mutant (*fliC* encodes the KLH11 flagellin) formed more robust biofilms compared the *fliC* mutant, suggesting that the KLH11 SsaRI system controls additional systems in addition to production of flagella that can affect biofilm formation [17].

Importantly and relevant to the environmental role for QS in roseobacters, active expression of *ssaI* and AHL molecules similar to those produced by SsaI are detected in whole extracts of wild-collected sponge tissue, which effectively connect findings on QS in the laboratory with the native host environment. Roseobacters are highly abundant in phytoplankton blooms or near macroalgae and are commonly found associated with marine invertebrates and organic particles. Motility is likely to play a critical role in many of these interactions. For example, mutants with motility defects in *Silicibacter* sp. TM1040 fail to attach to and form biofilms on host surfaces, specifically the dinoflagellate *Pfiesteria piscicida* [45], probably because TM1040 cannot chemotaxis to metabolites produced by the host, such as DMSP. In contrast, the KLH11 genome lacks any recognizable chemotaxis genes [30] and thus motility might play a different role in the symbiotic relationship with marine sponges. We hypothesize that the KLH11 QS pathway may play a role in dispersal. In nature, sponges actively pump large volumes (as many as 1000 L/h/kg of sponge tissue) of the surrounding seawater [7,8]. Given this tremendous flow rate microbial symbionts obtained from seawater at this stage likely do not require active motility to be introduced into the sponge host. Once KLH11-type bacteria colonize the sponge tissues and are provided a relatively nutrient rich environment, they may begin to grow to high density, perhaps beginning to aggregate. QS activation of motility and adherence inhibition may prevent aggregation in these crowded and potentially limiting microenvironments, and hence promote more uniform colonization of the host tissue or even stimulate release back into the water column. By coordinating motility and biofilm formation, motile KLH11 cells can mitigate against aggregation or even escape from their own aggregates. Experiments examining the colonization and distribution of KLH11 in live sponges, and tracking marked KLH11 QS mutants, may be the most direct approach to test these ecological hypotheses, and provide further insights into the environmental context of QS in this symbiotic system.

The proposed role in dispersal for KLH11 QS indeed resembles the role for QS in the water-borne pathogen *V. cholerae.* At low bacterial population densities such as when *V. cholerae* is in its aquatic reservoirs, it forms aggregates and biofilms. These aggregates and biofilms protect *V. cholerae* after ingestion into mammalian digestive tracts as they pass through the stomach into the intestine. The QS systems of *V. cholerae* (not AHLs but rather an alpha-hydroxy ketone and a furanosyl diester called autoinducer 2; both of which are chemically and mechanistically distinct from LuxR-LuxI systems) actively destabilize these biofilms in part through activation of motility and release motile, virulent cells into the intestinal environment where virulence factors such as cholera toxin can cause choleric dysentery. Explosive watery diarrhea releases large numbers of free-living *V. cholerae* into the aquatic environment where the cycle can re-initiate [46,47]. Furthermore, a similar dispersal model has also been proposed in *Rhodobacter sphaeroides* to prevent shading of photosynthetic activity, in which quorum sensing mutants form large aggregates [48].

## 6. QS in the Dinoflagellate Symbiont *D. shibae*

*D. shibae* was isolated from the dinoflagellate *Prorocentrum lima* [31] and lives symbiotically with its host marine alga. Genome sequence showed that it has three *luxI* homologues and both *luxI1* and *luxI2* are located on the chromosome and have a cognate *luxR* gene linked together while *luxI3* is located on a plasmid without a cognate *luxR* gene [32]. Expression of each of these three *luxI* type genes in *E. coli* resulted in production of AHLs. LuxI1 synthesized the primary AHL molecule C18en-HSL whereas LuxI2 and LuxI3 both synthesized long chain HSLs different from that of LuxI1. Interestingly, deletion of *luxI*1 in *D. shibae* resulted in complete loss of AHL synthesis and transcriptomic analysis showed that *luxI*1 can activate the expression of *luxI*2 and *luxI*3. In KLH11, the overall production of AHL is depressed in an *ssaI* mutant but the transcription of *ssbI or sscI* is not significantly affected, indicating a different control mechanism over the overall AHL production [33]. Most interestingly, *luxI*1 controls the heterogeneity of cell morphology in *D. shibae*. Wild type cultures exhibited heterogeneous cell morphology with respect to cell shape and size while LuxI1 mutant cultures were homogenous in size and morphology. *luxI*1 indeed controls cell cycle related genes such as *cckA*, *chpt* and *ctrA* but it is unknown how these genes might control the cell size and morphology [33]. The phenotypic variation of cell size and morphology controlled by QS in *D. shibae* has been described as risk-spreading or bet-hedging [49], processes which can potentially maximize the fitness of the bacterial population in fluctuating environments. In KLH11, *ssaIR* system transcriptionally controls expression of the *cckA-chpt-ctrA* pathway to activate flagellar motility [29]. In *D. shibae*, *luxI1* also activates expression of flagellar clusters and controls flagellar biosynthesis, and it seems likely that this is through *cckA-chpt-ctrA* similar to KLH11, but this has not been directly tested.

## 7. LuxI-LuxR Pathways in the *Roseobacter* Clade: A Bioinformatics Perspective

The first sequenced roseobacterial genome in the *Roseobacter* clade was *Ruegeria pomeroyi* DSS-3 (previously described as *Silicibacter pomeroyi* DSS-3) in 2004 [4]. Thus far, 57 roseobacterial genomes have been sequenced; including both finished and draft genomes that are deposited in public databases (see http://img.jgi.doe.gov/). Probing these 57 sequenced roseobacterial genomes using the SsaI and SsbI sequences of *Ruegeria* sp. KLH11 [17] and also the RhIL-type *luxI* homologue of *Roseobacter* sp. MED193 (Locus tag: MED193_08053) reveals that 49 (*ca.* 87%) of these genomes encode LuxI homologues. These 49 roseobacters include members from almost all the known *Roseobacter* genera, except for *Pelagibacter* and *Pseudovibrio*, although only one or two strains are sequenced from either of these two genera (Table 1). Interestingly, *Silicibacter* sp. TM1040 does not encode any *luxI* homologue whereas all the other sequenced close relatives within the *Silicibacter Ruegeria* subclade encode *luxI* homologues. In total, 89 *luxI* homologues were retrieved from these genomes using an *e* value < 10^−5^ (signifying the likelihood of the alignments occurring by chance) as the cutoff (Table S1).

Proteins of the LuxI family are usually 200 aa in length and are best conserved in their amino terminal region (1–100 aa) and more degenerate in their *C*-termini. There are generally eight invariant *N*-terminal residues among these enzymes. This conservation reflects crucial interactions with the common substrate *S*-adenosylmethionine (SAM) for the homoserine moiety through *N*-terminal residues and interaction with the more variable acyl-acyl carrier protein (acyl-ACP) through the *C*-terminus [50,51]. Sequence alignment of all the *Roseobacter* LuxI type sequences showed that the *N*-termini are well conserved (data not shown), with six out of the eight conserved residues perfectly conserved. These trends are consistent with the larger LuxI family and suggest that these eight sites are also critical for the LuxI-type enzyme activity in *Roseobacter* clade. As expected, the *C*-terminal region is more variable [18]. Several LuxI homologues have an extra long *C*-terminus, such as SsaI in *Ruegeria* sp. KLH11 (284 aa) and SiI1 in *R. pomeroyi* DSS-3 (286 aa, the longest LuxI homologue identified to date). However the majority of LuxI-type proteins in this *Roseobacter* clade range from 190 to 250 aa in length (Table S1).

Roughly thirty roseobacters encode multiple *luxI* homologues in their genomes; *Phaeobacter caeruleus* 13, DSM 24564 has the largest number of *luxI* homologues with five (Table S1). Phylogenetic analysis of these LuxI sequences indicates that *luxI* homologues in the same strains probably result from a combination of gene duplication and horizontal gene transfer (data not shown). For example, SsaI (RKLH11_3375) is closely related to SilI1 in *R. pomeroyi* DSS-3 while it only shares about 35% identity to SsbI (RKLH11_260) at the amino acid level [17]. Instead, SsbI shares 81% identity to SscI. Notably, SsbI shares 93% identity to a LuxI homologue in *Ruegeria* sp. TW15 (Locus tag: 010100017784). One possible explanation is that *sscI* does not have a cognate *luxR* homologue and thus might be subject to a higher evolution rate. It is commonly observed among diverse bacteria that *luxI* homologues are genetically linked to their cognate *luxR* homologues. However, 17 *luxI* homologues among the roseobacters were not linked to a *luxR* homologue and thus these are described as *luxI* solos. Interestingly, all the 7 roseobacters, which encode 3 *luxI* homologues, have a *luxI* solo, whereas 8 out of 19 genomes that encode two *luxI* homologues have solos, and even 2 out of 26 genomes that encode a single *luxI* homologue unlinked to a *luxR* homologue (Table S1).

## 8. New Structural Variants of AHL

**Figure 3.**
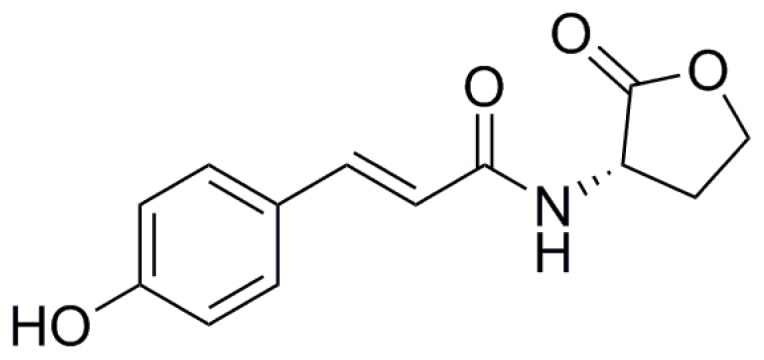
Structure of *p*-coumaroyl-HSL based on reference Schaefer *et al.* [52].

Recently, several novel types of signal molecules related to, but structurally distinct from AHLs have been reported [52–54]. The terrestrial and aquatic bacterium *Rhodopseudomonas palustris* produces *p-*coumaroyl-HSL (*p*C-HSL), incorporating a coumaroyl aryl group rather than the acyl chain of AHLs (Figure 3) [52]. Remarkably, *p*C-HSL is only produced in the presence of *p*-coumarate, a compound synthesized by plants and certain algae and directly incorporated into the signal molecule via the LuxI-type protein RpaI. *R. pomeroyi* DSS-3, was also found to produce this type of molecule, but also required growth on *p-*coumarate [52]. Bioassays using a *p*C-HSL responsive reporter strain of *R. palustris* that cannot synthesize *p*C-HSL but that directs RpaR-dependent expression of a target *rpaI-lacZ* fusion revealed that KLH11 also produces similar molecules in the presence of coumarate. Surprisingly, this was independent of any of the three known LuxI-type enzymes suggesting the existence of novel *Ruegeria* enzyme(s) responsible for *p*C-HSL synthesis in the presence of coumarate [40]. *Phaeobacter inhibens* DSM 17395 can also respond to the presence of *p*-coumarate produced by the microalga *Emiliania huxleyi* potentially via *p*C-HSL [55]. Furthermore *Silicibacter* sp. TM1040, which does not encode LuxI or LuxM homologues, produces the Roseobacter Motility Inducer (RMI) and this is stimulated by the presence of *p*-coumarate [56]. It is not known whether this is due to *p*C-HSL or some other compound. It is however clear that several roseobacters respond in complex ways to the presence of *p*-coumarate and this may drive intercellular signaling.

## 9. QS Control of Virulence Factors and Secondary Metabolites

Several roseobacterial strains are associated with diseases in marine invertebrates and algae. For example, *Roseovarius crassostreae* can infect hatchery-raised Eastern oysters (*Crassostrea virginica*) and cause seasonal mortalities in the northeastern United States [57,58]. *Nautella* sp. R11 (previously known as *Ruegeria* sp. R11) and *Phaeobacter* sp. LSS9 can cause a bleaching disease in the temperate-marine red macroalga, *Delisea pulchra* [59]. QS is well known to control the production of virulence factors in many different bacterial pathogens such as *V. cholerae*, *V. harveyi*, and *P. aeruginosa* [9]. An interesting link was proposed between QS and the production of virulence factors in the algal pathogens *Nautella* sp. R11 and *Phaeobacter* sp. LSS9. Both strains have two sets of LuxR-LuxI regulators and one additional *luxR* solo without a linked *luxI* homologue. Genomic comparisons between strains of *Nautella* and *Phaeobacter* which cause algal bleaching and related isolates that do not cause bleaching, reveals that the presence of specific *luxR* homologues correlates with the ability to cause bleaching [59]. However, additional experiments are required to establish a link between QS and virulence in these algal pathogens.

Roseobacters are also known to generate a variety of secondary metabolites [55,60,61]. QS might play a role in regulating the production of some of the metabolites, such as in *P. inhibens* DSM17395 as mentioned above [28]. Furthermore, *Phaeobacter* sp. Strain Y41 produces indigiodine via a nonribosomal peptide synthase and QS systems have been implicated in its control [61].

## 10. Future Directions

An increasing number of roseobacterial genome sequences are revealing the potential for extensive and diverse QS mechanisms. However, the regulatory networks that they orchestrate have only been studied in detail for a few specific species. Future research should focus on establishing more representative roseobacters, isolated from different ecological niches and representing a range of diversity, such as symbionts of algae and coral, for the study of their QS regulatory circuits, the AHLs and other signals they employ, and the output phenotypes that are controlled by QS. Better characterization of the LuxR-type protein target DNA binding sites, if they were well conserved, would help to identify genes that are controlled by QS using bioinformatics tools. As of yet, there is only one validated example of LuxR-type protein binding site that has been experimentally defined [17].

A major question remains regarding the ecological role of these diverse QS pathways in natural environments. A high level of detail has been revealed regarding the molecular mechanisms that control the symbiotic relationship between *V. fischeri* and its hosts, such as the Hawaiian bobtail squid *Euprymna scolopes.* The extensive understanding of this system has relied on the fact that the host *E. scolopes* can be readily grown and reproduced under laboratory conditions and that powerful genetic techniques exit to manipulate *V. fischeri*. The squid light organ is a highly closed environment that establishes a monoculture of *V. fischeri* [62]. The roseobacterial symbionts that utilize quorum sensing face a much more open system, with a great diversity of microbial co-residents and much greater flux from the environment. It would therefore be extremely valuable to establish a parallel model to study the symbiosis between roseobacters and their hosts, which represents such an open system relative to the intimate *V. fischeri*-squid symbiosis. As discussed above, roseobacters also clearly represent a potentially important pool for discovering novel QS molecules. Thus for a diversity of reasons, further exploration of the QS process in roseobacters is worthwhile and well warranted.

## 11. Conclusions

Acyl-homoserine lactone-based QS is widespread in members of the *Roseobacter* clade, with over 80% of available roseobacterial genomes encoding at least one *luxI* homologue. The three systems discussed here, *P. inhibens*, the sponge symbiont *Ruegeria* sp. KLH11, and the dinoflagellate symbiont *D. shibae*, provide some insights into the ecological roles of QS in this important clade of marine bacteria. Further examination of QS and the complex regulatory networks controlled by this signaling process in a greater diversity of roseobacters is needed to more fully elucidate the ecological roles of these diverse QS mechanisms.

## Supplementary Information



## Figures and Tables

**Table 1.** Summary of the three roseobacterial species reviewed.

Species	LuxI-LuxR	Isolation source	Signal profile	Function	Ref
*P. inhibens* DSM17395	*pgaRI*	coastal water	3-OH-C10-HSL	Temporal regulation of TDA production	[25–28]
*Ruegeria* sp. KLH11	*ssaRI*, *ssbRI sscI*	marine sponge *Mycale laxissima*	3-OH-C14-HSL, 3-OH-C14:1-HSL, 3-OH-C12-HSL	Activation of flagellar synthesis and swimming motility and inhibition of biofilm formation	[17,29,30]
*D. shibae* DFL12^T^	*luxR*1*I*1, *luxR*2*I*2, *luxI*3	Dinoflagellate *Prorocentrum lima*	3-C18en-HSL	Cell morphology and flagellar biosynthesis	[31–33]
