# Supplementary material for: Acyl-Homoserine Lactone Quorum Sensing in the Roseobacter Clade
Source: Int J Mol Sci. 2014 Jan 7;15(1):654–69. doi: 10.3390/ijms15010654 (PMC3907830; doi:10.3390/ijms15010654)
Supplement: Supplementary file 1 [file ijms-15-00654-s001.pdf]

Supplementary Information

Table S1. Summary of bioinformatic survey of LuxI homologues in sequenced roseobacter genomes.

Locus Tag [1,2]	LuxI AA Seq Length	Species
C357_10192	211	*Citreicella* sp. 357
CSE45_4054	211	*Citreicella* sp. SE45
**Dshi_4152**	211	*Dinoroseobacter shibae* DFL-12, DSM 16493
Dshi_2851	206	*Dinoroseobacter shibae* DFL-12, DSM 16493
Dshi_0312	195	*Dinoroseobacter shibae* DFL-12, DSM 16493
Jann_0620	195	*Jannaschia* sp. CCS1
Leiaq_00074	213	*Leisingera aquimarina* DSM 24565 (scaffold version)
Leiaq_04264	190	*Leisingera aquimarina* DSM 24565 (scaffold version)
Leime_1139	213	*Leisingera methylohalidivorans* MB2, DSM 14336
Leime_2648	221	*Leisingera methylohalidivorans* MB2, DSM 14336
Leina_00212	266	*Leisingera nanhaiensis* NH52F, DSM 24252 (scaffold version)
Leina_03458	212	*Leisingera nanhaiensis* NH52F, DSM 24252 (scaffold version)
LSE62_0617	211	*Loktanella* sp. SE62
LSE62_3231	224	*Loktanella* sp. SE62
H147DRAFT_00614	224	*Loktanella vestfoldensis* DSM 16212
SKA53_05830	212	*Loktanella vestfoldensis* SKA53
RB2654_09014	213	*Maritimibacter alkaliphilus* HTCC2654
RB2654_20048	195	*Maritimibacter alkaliphilus* HTCC2654
B139DRAFT_0037	195	*Maritimibacter* sp. HL-12
**B139DRAFT_2385**	215	*Maritimibacter* sp. HL-12
RR11_2017	200	*Nautella italica* R11
RR11_2520	213	*Nautella italica* R11
**OIHEL45_00955**	279	*Oceanibulbus indolifex* HEL-45
OG2516_02294	214	*Oceanicola granulosus* HTCC2516
OCGS_2461	196	*Oceaniovalibus guishaninsula* JLT2003
OAN307_c21810	208	*Octadecabacter antarcticus* 307
OAN307_c41320	212	*Octadecabacter antarcticus* 307
OA238_2886	134	*Octadecabacter arcticus* 238, DSM 13978
Phaar_01741	193	*Phaeobacter arcticus* DSM 23566
Phaar_02924	213	*Phaeobacter arcticus* DSM 23566
**Phaar_00769**	200	*Phaeobacter arcticus* DSM 23566
PhacaeDRAFT_0324	190	*Phaeobacter caeruleus* 13, DSM 24564
PhacaeDRAFT_1367	213	*Phaeobacter caeruleus* 13, DSM 24564
PhacaeDRAFT_2710	221	*Phaeobacter caeruleus* 13, DSM 24564
PhacaeDRAFT_3752	208	*Phaeobacter caeruleus* 13, DSM 24564
PhacaeDRAFT_4672	212	*Phaeobacter caeruleus* 13, DSM 24564
Phada_01951	213	*Phaeobacter daeponensis* TF-218, DSM 23529 (scaffold version)
Phada_03339	221	*Phaeobacter daeponensis* TF-218, DSM 23529 (scaffold version)
**PGA1_c07680**	202	*Phaeobacter gallaeciensis* DSM 17395
PGA2_c03440	213	*Phaeobacter gallaeciensis* 2.10
**PGA2_c07460**	249	*Phaeobacter gallaeciensis* 2.10

Table S1. *Cont.*

**Locus Tag** [1,2]	**LuxI AA Seq Length**	**Species**
PGA2_c18960	235	*Phaeobacter gallaeciensis* 2.10
RGBS107_08956	213	*Phaeobacter gallaeciensis* DSM 17395
PhainDRAFT_0507	235	*Phaeobacter inhibens* T5, DSM 16374
PhainDRAFT_1820	190	*Phaeobacter inhibens* T5, DSM 16374
PhainDRAFT_2120	213	*Phaeobacter inhibens* T5, DSM 16374
**PhainDRAFT_1688**	202	*Phaeobacter inhibens* T5, DSM 16374
RBY41_3464	221	*Phaeobacter* sp. Y41.
RBY41_3631	213	*Phaeobacter* sp. Y41.
ANG1_1315	213	*Phaeobacter gallaeciensis* ANG1
D516_1373	*210*	*Rhodobacter sp. AKP1*
RB2083_730	207	Rhodobacterales sp. HTCC2083
RB2083_3255	259	Rhodobacterales sp. HTCC2083
RB2150_11291	213	Rhodobacterales sp. HTCC2150
RB2150_14421	205	Rhodobacterales sp. HTCC2150
RD1_1639	212	*Roseobacter denitrificans* OCh 114
RLO149_c030680	212	*Roseobacter litoralis* Och 149
RLO149_c036210	210	*Roseobacter litoralis* Och 149
**RLO149_c036590**	214	*Roseobacter litoralis* Och 149
RAZWK3B_04275	212	*Roseobacter* sp. AzwK-3b
**RAZWK3B_19371**	248	*Roseobacter* sp. AzwK-3b
MED193_10423	213	*Roseobacter* sp. MED193
**MED193_08053**	200	*Roseobacter* sp. MED193
R2A57_2404	212	*Roseobacter* sp. R2A57
**RSK20926_22084**	221	*Roseobacter* sp. SK209-2-6
RSK20926_15131	246	*Roseobacter* sp. SK209-2-6
RCCS2_02078	224	*Roseobacter* sp. CCS2
ROS217_18267	264	*Roseovarius* sp. 217
ROS217_01410	221	*Roseovarius* sp. 217
RTM1035_10485	264	*Roseovarius* sp. TM1035
**ISM_03755**	201	*Roseovarius nubinhibens* ISM
SPO0372	212	*Ruegeria pomeroyi* DSS-3
SPO2287	286	*Ruegeria pomeroyi* DSS-3
RKLH11_260	212	*Ruegeria* sp. KLH11
RKLH11_2275	284	*Ruegeria* sp. KLH11
**RKLh11**	214	*Ruegeria* sp. KLH11
**SCH4B_1938**	210	*Silicibacter* sp. TrichCH4B
**RTW15_010100005486**	200	*Ruegeria* sp. TW15
RTW15_010100013872	284	*Ruegeria* sp. TW15
RTW15_010100017784	212	*Ruegeria* sp. TW15
SSE37_11164	212	*Sagittula stellata* E-37
SL1157_0612	212	*Ruegeria lacuscaerulensis* ITI-1157
SL1157_1706	221	*Ruegeria lacuscaerulensis* ITI-1157
SL1157_2476	284	*Ruegeria lacuscaerulensis* ITI-1157

Table S1. *Cont.*

Locus Tag [1,2]	LuxI AA Seq Length	Species
**EE36_01635**	242	*Sulfitobacter* sp. EE-36
**RGAI101_1101**	248	*Sulfitobacter* sp. GAI101
RGAI101_3395	211	*Sulfitobacter* sp. GAI101
**NAS141_00695**	242	*Sulfitobacter* sp. NAS-14.1
NAS141_01136 (is not used for tree thus 96seuqneced)	121	*Sulfitobacter* sp. NAS-14.1
TR2A62_3166	220	*Thalassiobium* sp. R2A62
		Loktanella hongkongensis DSM 17492 [3]
		Oceanicola batsensis HTCC2597 [3]
		Pelagibaca bermudensis HTCC2601 [3]
		Rhodobacterales sp. HTCC2255 [3]
		Ruegeria sp. TM1040 [3]
		Pseudovibrio sp. JE062 [3]
		Pseudovibrio sp. FO-BEG1 [3]

[1] All the homologues belong to the family of Pfam00765; [2] Bold indicates LuxI solo; [3] The sequenced genomes that do not have LuxI homologue.

© 2014 by the authors; licensee MDPI, Basel, Switzerland. This article is an open access article distributed under the terms and conditions of the Creative Commons Attribution license (http://creativecommons.org/licenses/by/3.0/).
